# Large Language Models and Text Embeddings for Detecting Depression and Suicide in Patient Narratives

**DOI:** 10.1001/jamanetworkopen.2025.11922

**Published:** 2025-05-23

**Authors:** Silvia Kyungjin Lho, Sang-Cheol Park, Hahyun Lee, Da Young Oh, Hyeonjin Kim, Soomin Jang, Hee Yeon Jung, So Young Yoo, Su Mi Park, Jun-Young Lee

**Affiliations:** 1 Department of Psychiatry, Seoul Metropolitan Government–Seoul National University Boramae Medical Center, Seoul, Republic of Korea; 2 Institute for Biomaterials, Korea University, Seoul, Republic of Korea; 3 Interdisciplinary Program in Cognitive Science, Seoul National University, Seoul, Republic of Korea; 4 Department of Psychiatry, Seoul National University College of Medicine, Seoul, Republic of Korea; 5 Department of Counseling Psychology, Hannam University, Daejeon, Republic of Korea

## Abstract

**Importance:**

Large language models (LLMs) and text-embedding models have shown potential in assessing mental health risks based on narrative data from psychiatric patients.

**Objective:**

To assess whether LLMs and text-embedding models can identify depression and suicide risk based on sentence completion test (SCT) narratives of psychiatric patients.

**Design, Setting, and Participants:**

This cross-sectional study, conducted at Seoul Metropolitan Government-Seoul National University Boramae Medical Center, analyzed SCT data collected from April 1, 2016, to September 30, 2021. Participants included psychiatric patients aged 18 to 39 years who completed SCT and self-assessments for depression (Beck Depression Inventory–II or Zung Self-Rating Depression Scale) and/or suicide (Beck Scale for Suicidal Ideation). Patients confirmed to have an IQ below 70 were excluded, leaving 1064 eligible SCT datasets (52 627 completed responses). Data processing with LLMs (GPT-4o, May 13, 2024, version; OpenAI [hereafter, *LLM1*]; gemini-1.0-pro, February 2024 version; Google DeepMind [hereafter, *LLM2*]; and GPT-3.5-turbo-16k, January 25, 2024, version; OpenAI) and text-embedding models (text-embedding-3-large, OpenAI [hereafter, *text-embedding 1*]; text-embedding3-small; OpenAI; and text-embedding-ada-002; OpenAI) was performed between July 4 and September 30, 2024.

**Main Outcomes and Measures:**

Outcomes included the performance of LLMs and text-embedding models in detecting depression and suicide, as measured by the area under the receiver operating characteristic curve (AUROC), balanced accuracy, and macro F1-score. Performance was evaluated across concatenated narratives of SCT, including self-concept, family, gender perception, and interpersonal relations narratives.

**Results:**

Based on SCT narratives from 1064 patients (mean [SD] age, 25.4 [5.5] years; 673 men [63.3%]), LLM1 showed strong performance in zero-shot learning, with an AUROC of 0.720 (95% CI, 0.689-0.752) for depression and 0.731 (95% CI, 0.704-0.762) for suicide risk using self-concept narratives. Few-shot learning for depression further improved the performance of LLM1 (AUROC, 0.754 [95% CI, 0.721-0.784]) and LLM2 (AUROC, 0.736 [95% CI, 0.704-0.770]). The text-embedding 1 model paired with extreme gradient boosting outperformed other models, achieving an AUROC of 0.841 (95% CI, 0.783-0.897) for depression and 0.724 (95% CI, 0.650-0.795) for suicide risk. Overall, self-concept narratives showed the most accurate detections across all models.

**Conclusions and Relevance:**

This cross-sectional study of SCT narratives from psychiatric patients suggests that LLMs and text-embedding models may effectively detect depression and suicide risk, particularly using self-concept narratives. However, while these models demonstrated potential for detecting mental health risks, further improvements in performance and safety are essential before clinical application.

## Key Points

**Question:**

Can large language models (LLMs) and text-embedding models detect depression and suicide risk based on sentence completion test (SCT) narratives of psychiatric patients?

**Findings:**

In this cross-sectional study of SCT datasets from 1064 patients (52 627 completed responses), both LLMs and text-embedding models showed strong performance, with areas under the receiver operating characteristic curve greater than 0.7 in detecting clinically significant depression and high risk of suicide, particularly based on self-concept narratives.

**Meaning:**

This study suggests that LLMs and text-embedding models have potential to detect mental health risks, including depression and suicide, but further performance improvement and addressing ethical concerns are essential for clinical application.

## Teaser

This cross-sectional study assesses whether large language models (LLMs) and text-embedding models can detect clinically significant depression and high risk of suicide based on sentence completion test narratives of psychiatric patients.

## Introduction

After the release of Chat Generative Pre-trained Transformer (ChatGPT) in November 2022, ChatGPT has demonstrated capabilities such as passing the United States Medical Licensing Examination^1,2,3^ and excelling in clinical reasoning and diagnosing across various medical fields.^4,5,6,7,8,9^ The psychiatric field has emerged as a particularly promising area for the implementation of artificial intelligence (AI) models, especially large language models (LLMs), because psychiatric evaluation relies heavily on qualitative and nuanced verbal narratives provided by patients.^10,11^

To date, natural language processing (NLP) and machine learning (ML) techniques have proven value in extracting specific key words related to psychiatric symptoms from patients’ narratives, aiding in diagnostic evaluation.^12^ The evolution from traditional NLP to modern LLMs represents a natural progression in this field, leading to sophisticated applications. For example, domain-specific ML models, such as MentalBERT,^13^ have been developed for detecting stress, depression, or suicide risk based on social media content.^13,14,15,16^ More recently, researchers have explored the capabilities of LLMs in detecting depression and suicidal tendencies using online data.^17,18^ Notably, Bartal et al^19^ used Generative Pre-trained Transformer 3.5 (GPT-3.5 [OpenAI]) and text-embedding models to identify childbirth-related posttraumatic stress disorder based on childbirth narratives, showing potential of LLMs for mental health risk assessment.

Despite these advancements, a gap remains in applying the latest language models to detect mental health risks based on patients’ narratives in clinical settings. Although domain-specific pretraining has been considered necessary for strong performance, the primary challenge may lie in the narrative quality and structure rather than model specialization. Properly structured and targeted narratives could enable general-purpose language models to effectively screen for mental health risks. The cognitive triad of Beck,^20^ which suggests that individuals with depression possess negative views of the self, future, and world, supports the idea that patients’ narratives can serve as a basis for diagnostic evaluation. In this context, the sentence completion test (SCT), a projective test developed for assessing intelligence and personality,^21,22^ offers a unique opportunity providing more targeted narratives by eliciting attitudes toward the self, others, and the world. Although SCT use has declined in Western countries following trends toward evidence-based diagnoses,^23,24,25,26^ it remains popular in Asia, including South Korea,^25,26,27^ valued for uncovering hidden thoughts and attitudes critical to psychiatric diagnosis.^21,22^

This study investigates whether LLMs and text-embedding models can detect clinically significant depression and high risk of suicide based on SCT narratives. We aim to assess general-purpose models with prompt engineering, eliminating the need for domain-specific pretraining, while using text-embedding models to extract vectors for training ML models tailored to our dataset. We hypothesize that LLMs and text-embedding models can analyze SCT narratives to detect depression and suicide risk. In addition, we hypothesize that specific topics, such as self-concept narratives, may provide more targeted information aligned with Beck’s cognitive triad of depression.

## Methods

Procedures for this cross-sectional study were approved by the institutional review board of the Seoul Metropolitan Government–Seoul National University Boramae Medical Center. Informed consent was waived by the institutional review board as the study had a retrospective design and used anonymized clinical data. We followed the Strengthening the Reporting of Observational Studies in Epidemiology (STROBE) reporting guideline for cross-sectional studies.

### Data Source

We conducted a retrospective observational study as part of an evaluation of the efficiency of the diagnosis and treatment prognosis of psychological assessment at the Seoul Metropolitan Government–Seoul National University Boramae Medical Center. Psychiatric patients aged 18 to 39 years, who completed psychological evaluation between April 1, 2016, and September 30, 2021, were included. Patients were excluded if they lacked self-assessment depression scores (the Korean version of the Beck Depression Inventory–II [K-BDI-II] or Zung Self-Rating Depression Scale [SDS]), had incomplete SCT items (missing more than one-third of items), or had a confirmed Full Scale Intelligence Quotient (FSIQ) below 70.

### Clinical Measures and Defining Mental Health Risk

The SCT, a semiprojective psychological test, involves completing a series of incomplete sentences to reflect an individual’s self-concept and attitudes toward various aspects.^21^ The Sacks Sentence Completion Test (SSCT), the most widely used version,^21,22^ was adapted for a Korean version of the SSCT, consisting of 50 items categorized into 4 areas: self-concept (16 items), family (10 items), gender perception (15 items), and interpersonal relations (9 items).^22^ A translated version of the Korean SCT is presented in eTable 1 in Supplement 1. Items in each area were concatenated into 4 narratives for each participant.

Depression was assessed using the K-BDI-II or SDS. The K-BDI-II^28,29^ is a 21-item scale (total score, 0-63; 0-13 indicates indicating minimal depression, 14-19 indicates mild depression, 20-28 indicates moderate depression, and 29-63 indicates severe depression. The SDS^30,31^ is a 20-item scale (total score, 25-100; where 25-49 indicates no depression, 50-59 indicates mild depression, 60-69 indicates moderate to marked depression, and 70-100 indicates severe to extreme major depression. We used the cutoff values for moderate depression of both scales to define clinically significant depression.

**Figure 1.**
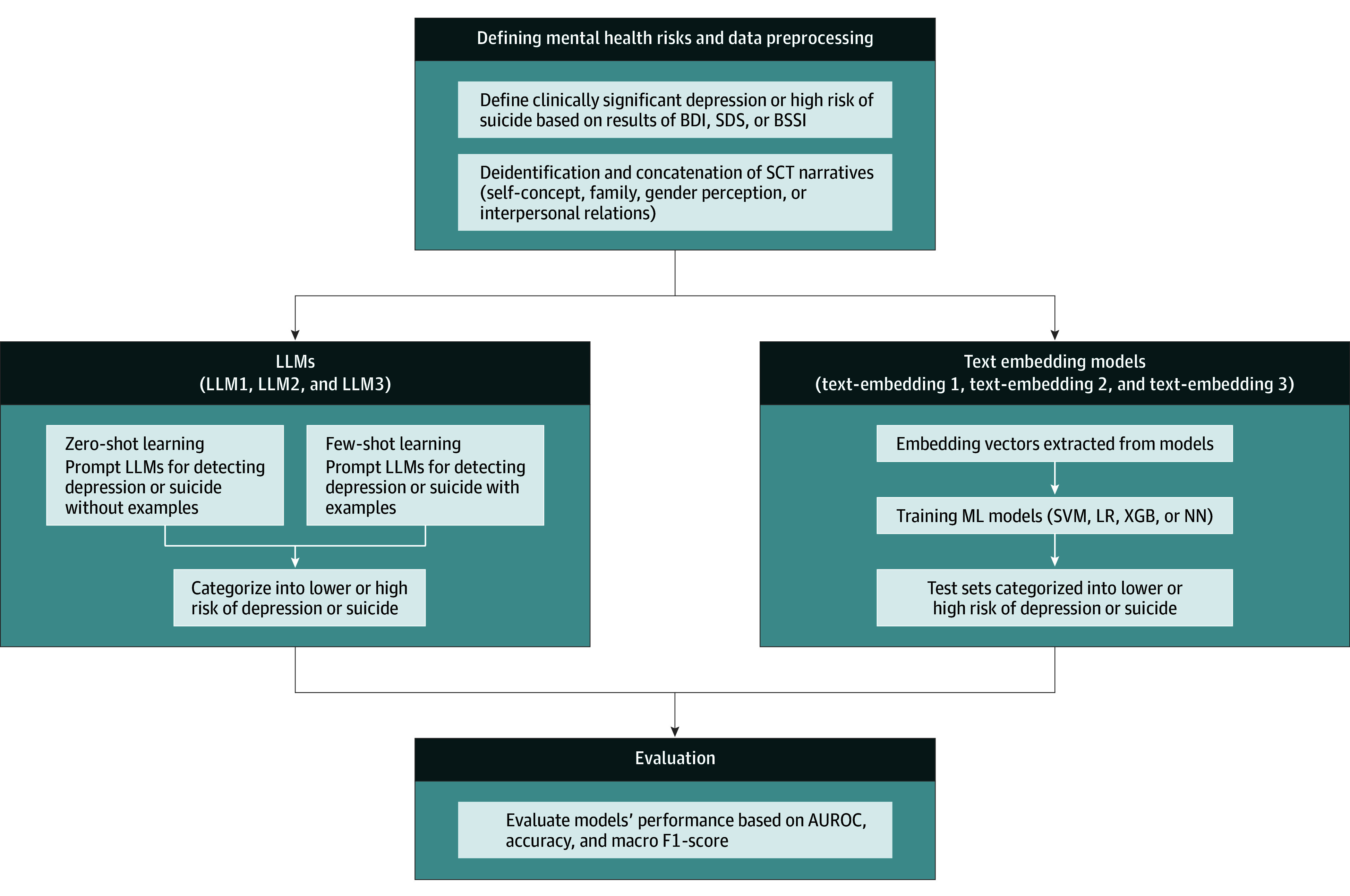
Workflow for Depression and Suicide Risk Detection Using Large Language Models (LLMs) and Text-Embedding Models Preprocessing involved concatenating sentence completion test (SCT) narratives and defining clinically significant depression or high risk of suicide using Beck Depression Index (BDI) and/or Zung Self-Rating Depression Scale (SDS) or Beck Suicidal Severity Index (BSSI). Detecting mental health risks included the use of large language models (LLMs) and embedding-based machine learning (ML) models (support vector machine [SVM], logistic regression [LR], extreme grading boosting [XGB], and neural network [NN]). The evaluation process assesses model performance using area under the receiver operating characteristic curve (AUROC), accuracy, and macro F1-score. LLM1 indicates GPT-4o (May 13, 2024, version; OpenAI); LLM2, gemini-1.0-pro (February 2024 version; Google); LLM3, GPT-3.5-turbo-16k (January 25, 2024, version; OpenAI); text-embedding 1, text-embedding-3-large (OpenAI), text-embedding 2, text-embedding-3-small (OpenAI), and text-embedding 3, text-embedding-002-ada (OpenAI).

The Korean version of the Beck Scale for Suicidal Ideation is a 19-item self-assessment scale evaluating suicidal ideation over the past week, with scores ranging from 0 to 38, where higher scores indicate greater suicide risk.^32,33,34^ Although the original Beck Scale for Suicidal Ideation developers did not establish a validated cutoff score,^33^ several studies have proposed practical cutoff values.^34,35,36^ Based on Korean validation studies,^37^ we used a score of 15 or higher to define high risk of suicide. The FSIQ was assessed using the Korean Wechsler Adult Intelligence Scale, Fourth Edition (K-WAIS-IV),^38^ which is the Korean adaption of the original WAIS-IV.^39^ The FSIQ is a composite score of multiple subtests, with scores ranging from 40 to 160, where higher scores indicate greater cognitive ability. The workflow of the study is shown in Figure 1.

### Data Preprocessing

To protect patient privacy, SCT narratives were deidentified before being processed by LLMs and text-embedding models, following the Health Insurance Portability and Accountability Act guidelines.^40^ A manual review of all 50 items across SCT data identified 42 responses containing patient or relative names in 41 SCT datasets. We developed an NLP-based system using the MeCab (Python Software Foundation) tokenizer for Korean language, flagging proper nouns (2-4 characters) through part-of-speech tagging and noun extraction. In addition, the system identified sensitive information such as dates and numeric identifiers. From 720 flagged cases, manual review confirmed 19 sensitive responses (18 names and 1 birth date) in 14 SCT data items. All 61 responses were deidentified through pseudonymization and generalization. Reidentification risk was assessed by designating the 4 concatenated narratives as quasi-identifiers and analyzing them with TF-IDF (term frequency–inverse document frequency) vectors and pairwise cosine distances using k-nearest neighbor analysis (k = 5). Manual review of flagged records confirmed minimal reidentification risk. Python, version 3.10.12 (Python Software Foundation), was used for data preprocessing and all subsequent analyses, except for specified statistical analyses.

### Detecting Mental Health Risk Using LLMs and Text-Embedding Models

Following the methodology of Bartal et al,^19^ we evaluated leading LLMs with zero-shot and few-shot learning as well as embedding-based ML models in detecting depression and suicide risk. For LLM evaluation, we used GPT-4o (May 13, 2024, version; OpenAI [hereafter, *LLM1*]), gemini-1.0-pro (February 2024 version; Google [hereafter, *LLM2*]), and GPT-3.5-turbo-16k (January 25, 2024, version; OpenAI [hereafter, *LLM3*]). GPT-4 (June 13, 2024, version) and gemini-1.5-pro (May 2024 version) were excluded due to higher costs and lower performance in the preliminary evaluation. For zero-shot learning, we adapted the prompt from previous studies,^18,19^ as shown in Table 1, with temperature set to 0 for deterministic responses. In few-shot learning, we provided 4 narrative examples for each task: 2 from participants with clinically significant depression and 2 with subclinical or no depression for the depression task, and 2 from high-risk and 2 from low-risk participants for the suicide task, along with their labels. In preliminary testing, 1 narrative per class showed lower performance, while more than 2 had no additional benefit. Thus, we selected 2 narratives per class for cost-effectiveness. Model performance was evaluated using the area under the receiver operating characteristic curve (AUROC), accuracy, and macro F1-score, all reported with 95% CIs.

**Table 1.** Prompts Used for Zero-Shot and Few-Shot Learning for LLM1, LLM2, and LLM3

Mental health risk and category	Prompts
Depression	
Zero-shot	You are an experienced psychiatrist specializing in diagnosing and treating depression. I will provide you with narratives from a participant’s sentence completion test (SCT). Your task is to decide whether the participant is at high risk of depression (Label 1) or lower risk of depression (Label 0). Provide only ‘1’ or ‘0’ based on a reasonable explanation.
Few-shot	You are an experienced psychiatrist specializing in diagnosing and treating depression. I will provide you with narratives from a participant’s sentence completion test (SCT). Your task is to decide whether the participant is at high risk of depression (Label 1) or lower risk of depression (Label 0). Provide only ‘1’ or ‘0’ based on a reasonable explanation. Here are a few examples of narratives and their labels. ###<Narrative>: example<Label>: 1 ###<Narrative>: example<Label>: 0
Suicide	
Zero-shot	You are an experienced psychiatrist specializing in diagnosing and treating suicidal patient. I will provide you with narratives from a participant’s sentence completion test (SCT). Your task is to decide whether the participant is at high risk of suicide (Label 1) or lower risk of suicide (Label 0). Provide only ‘1’ or ‘0’ based on a reasonable explanation.
Few-shot	You are an experienced psychiatrist specializing in diagnosing and treating suicidal patient. I will provide you with narratives from a participant’s sentence completion test (SCT). Your task is to decide whether the participant is at high risk of suicide (Label 1) or lower risk of suicide (Label 0). Provide only ‘1’ or ‘0’ based on a reasonable explanation. Here are a few examples of narratives and their labels. ###<Narrative>: example<Label>: 1 ###<Narrative>: example<Label>: 0

Abbreviations: LLM1, GPT-4o (May 13, 2024, version; OpenAI); LLM2, gemini-1.0-pro (February 2024 version; Google); LLM3, GPT-3.5-turbo-16k (January 25, 2024, version; OpenAI).

For embedding-based ML models, we used OpenAI’s text-embedding models (text-embedding-3-large [hereafter, *text-embedding 1*], text-embedding-3-small [hereafter, *text-embedding 2*], and text-embedding-002-ada [hereafter, *text-embedding 3*]) to extract embeddings with dimensions of 3072, 1536, and 1536, respectively. After splitting the data into training and testing sets (80:20) and applying standard scaling, we trained support vector machines (SVMs) and logistic regression (LR) using Scikit-learn (Python),^41^ and extreme gradient boosting (XGB) models using XGBoost^42^ with the class weight set to “balanced” to handle class imbalances. Each model underwent 5-fold stratified cross-validation and was evaluated on the test set for the AUROC, accuracy, and macro F1-score. In addition, we implemented a neural network (NN) using TensorFlow/Keras^43^ with 3 hidden layers (256, 128, and 64 units), dropout layers, and L2 regularization to prevent overfitting. The network was compiled with the Adam optimizer and trained with early stopping on the scaled embedding vectors. The binary cross-entropy loss was used for training. The NN was evaluated on the same metrics as the other ML models.

### Statistical Analysis

Data processing with LLMs and text-embedding models was performed between July 4 and September 30, 2024. Demographic and clinical characteristics between groups were compared using an independent *t* test for continuous variables and χ^2^ analysis for the categorical data. All *P* values were from 2-sided tests and results were deemed statistically significant at *P* < .05. Analyses were conducted using IBM SPSS, version 23 (IBM Corp). The AUC scores of LLMs, evaluated on the entire dataset, were compared separately from those of embedding-based ML models, evaluated on a test dataset. Because probability scores were unavailable for the LLMs, whose detections were associated with the prompt when making detections, we used bootstrapping with 1000 resamples to estimate 95% CIs for AUROC scores. In each iteration, we randomly sampled the dataset with replacement, maintaining the original sample size, and calculated the AUROC. The 95% CI was derived from the 2.5th and 97.5th percentiles of the AUROC distribution. We then calculated mean AUROC differences between models. Statistical significance was determined by whether the 95% CI of the mean AUROC difference included zero. The Cochran *Q* test assessed overall differences in accuracy between LLMs, followed by McNemar tests with Bonferroni correction for pairwise comparison (*P* < .05/15, comparing 6 models) using IBM SPSS, version 23.

We performed qualitative analysis on 3 representative cases including 4 narratives to examine factors associated with model performance. Using a comparative case analysis approach, we examined narratives in which LLM1 either detected or failed to detect depression. We analyzed thematic content and psychological characteristics to identify factors possibly associated with model performance. A modified prompt was used to elicit the reasoning process of LLM1. Detailed methods, specific prompt, and narratives are provided in the eMethods and eTable 9 in Supplement 1.

## Results

### Patient Characteristics

From the initial 2443 patients, 1532 patients participated in SCTs. We excluded 349 patients due to a lack of self-assessment depression scores or incomplete SCT items and 119 patients with an IQ below 70. This resulted in 1064 patients (mean [SD] age, 25.4 [5.5] years; 673 men [63.3%] and 391 women [36.7%]; mean [SD] education, 14.1 [1.9] years), with a total of 52 627 completed item responses across all patients for the final analysis (Table 2). Depression detection was conducted on all 1064 SCT datasets, while suicide risk assessment was performed on 882 SCT datasets.

**Table 2.** Patient Demographic and Clinical Characteristics

Characteristic	Depression				Suicide			
	Total (n = 1064)	No or subclinical depression (n = 254)	Clinically significant depression (n = 810)	*P* value^a^	Total (n = 882)	Low risk (n = 470)	High risk (n = 412)	*P* value^a^
Age, mean (SD), y	25.4 (5.5)	26.4 (6.1)	25.1 (5.3)	.003	25.6 (5.6)	26.2 (6.0)	24.9 (5.1)	<.001
Sex, No (%)								
Male	673 (63.3)	174 (68.5)	499 (61.6)	.05	556 (63.0)	299 (63.6)	257 (62.4)	.70
Female	391 (36.7)	80 (31.5)	311 (38.4)		326 (37.0)	171 (36.4)	155 (37.6)	
Education, mean (SD), y^b^	14.1 (1.9)	14.4 (1.9)	14.0 (1.9)	.004	14.1 (1.9)	13.9 (1.9)	14.2 (1.9)	.002
BDI-II score, mean (SD)^b^	28.3 (15.1)	10.0 (5.81)	35.5 (10.9)	<.001	29.0 (14.1)	26.7 (13.9)	38.0 (11.4)	.002
SDS score, mean (SD)^b^	68.5 (12.8)	50.2 (7.09)	73.9 (8.35)	<.001	68.8 (12.7)	62.0 (11.7)	75.7 (9.64)	<.001
BSSI score, mean (SD)^b^	13.2 (9.71)	5.71 (6.17)	15.4 (9.45)	<.001	13.2 (9.71)	5.43 (4.90)	22.2 (5.02)	<.001
FSIQ score, mean (SD)^b^	95.7 (14.7)	96.4 (14.7)	95.5 (14.7)	.87	95.9 (14.8)	96.6 (14.9)	95.2 (14.8)	.19

Abbreviations: BDI-II, Beck Depression Inventory–II; BSSI, Beck Scale for Suicidal Ideation; FSIQ, Full-Scale Intelligence Quotient; SDS, Zung Self-Rating Depression Scale.

^a^ Derived from an independent *t* test for continuous variables and a χ^2^ test for categorical variables.

^b^ Available data for each category: education (n = 1050 for depression, n = 868 for suicide), BDI-II (n = 232 for depression, n = 93 for suicide), SDS (n = 874 for depression and 830 for suicide), BSSI (n = 882 for both depression and suicide), FSIQ (n = 946 for depression, n = 782 for suicide) (see Methods for details).

### Overall Evaluations of the Models

**Figure 2.**
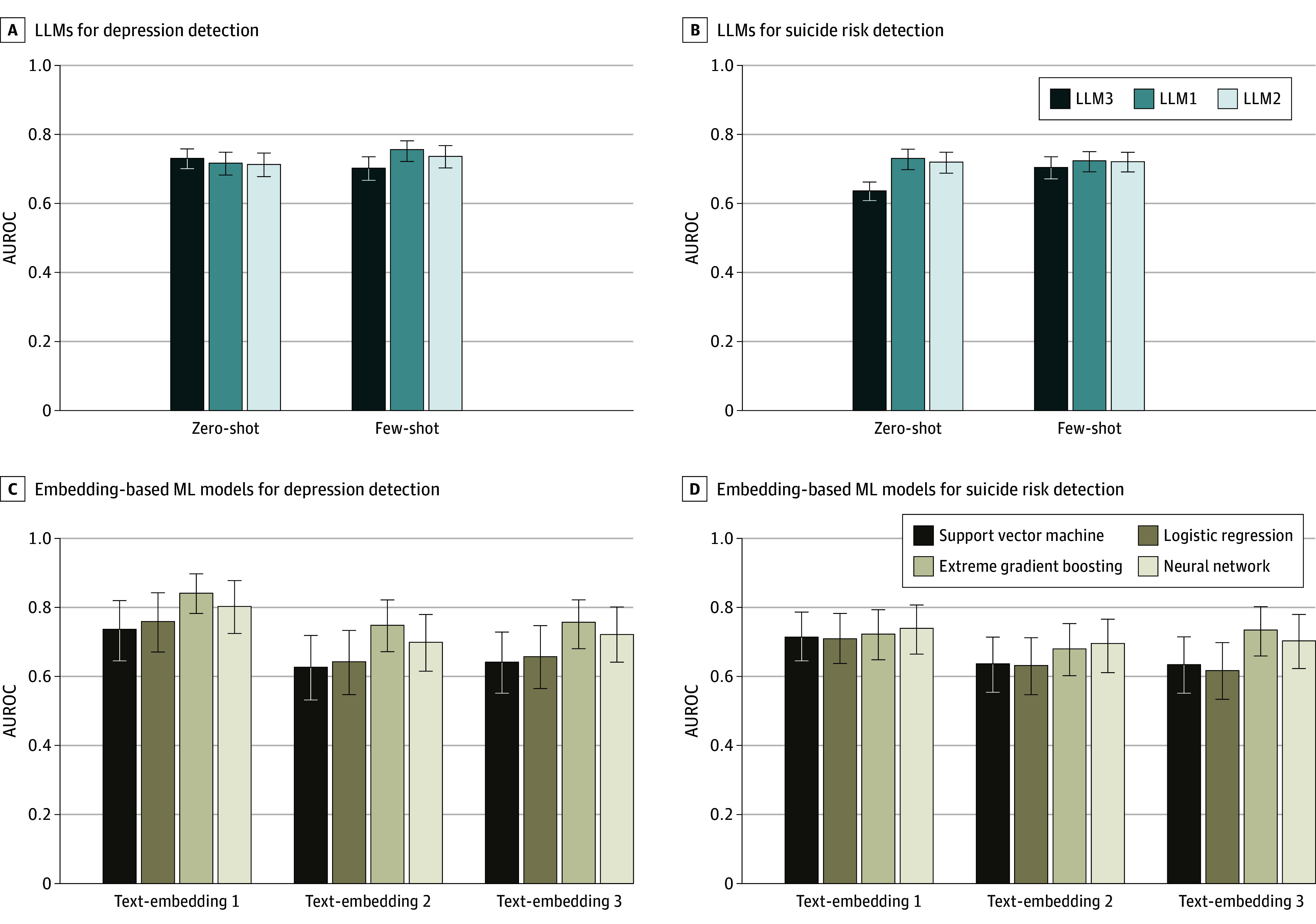
Comparison of Model Performance (Area Under the Receiver Operating Characteristic Curve [AUROC]) With 95% CIs A, Large language models (LLMs) for depression detection. B, LLMs for suicide risk detection. C, Embedding-based machine learning (ML) models for depression detection. D, Embedding-based ML models for suicide risk detection. LLM1 indicates GPT-4o (May 13, 2024, version; OpenAI); LLM2, gemini-1.0-pro (February 2024 version; Google); LLM3, GPT-3.5-turbo-16k (January 25, 2024, version; OpenAI); text-embedding 1, text-embedding-3-large (OpenAI), text-embedding 2, text-embedding-3-small (OpenAI), and text-embedding 3, text-embedding-002-ada (OpenAI).

The performance of LLMs and embedding-based ML models in detecting clinically significant depression and high risk of suicide was evaluated based on 4 narratives: self-concept, family, gender perception, and interpersonal relations (Table 3; Figure 2; eTables 2-4 in Supplement 1). Text-embedding 1 consistently outperformed smaller embeddings, although performance varied by narrative types. The analysis suggested that embedding-based ML models, particularly with XGB or NN, provided the best results for both detection tasks. These models outperformed the overall metrics of LLMs in detecting both depression and suicide, although direct statistical comparisons were unavailable. However, LLM1 and LLM2 showed strong AUROC performance, particularly in the few-shot learning. Across LLMs and embedding-based ML models, detections using self-concept narratives achieved the highest performance, with AUROCs of approximately 0.7 to 0.8, a range higher than those achieved with family, gender perception, or interpersonal relations narratives.

**Table 3.** Performance of Zero-Shot and Few-Shot LLMs and Embedding-Based Machine Learning Models Detecting Clinically Significant Depression and High Risk of Suicide Based on SCT Self-Concept Narratives

Category and model	Depression			Suicide		
	AUROC (95% CI)	Accuracy (95% CI)	Macro F1-score (95% CI)	AUROC (95% CI)	Accuracy (95% CI)	Macro F1-score (95% CI)
Zero-shot						
LLM1	0.720 (0.689-0.752)	0.814 (0.789-0.837)	0.730 (0.697-0.763)	0.731 (0.704-0.762)	0.734 (0.706-0.764)	0.731 (0.702-0.761)
LLM2	0.714 (0.680-0.747)	0.814 (0.789-0.836)	0.727 (0.693-0.757)	0.721 (0.695-0.750)	0.715 (0.686-0.743)	0.715 (0.686-0.743)
LLM3	0.731 (0.703-0.761)	0.677 (0.648-0.705)	0.650 (0.620-0.678)	0.635 (0.609-0.659)	0.654 (0.622-0.685)	0.611 (0.575-0.643)
Few-shot						
LLM1	0.754 (0.721-0.784)	0.745 (0.717-0.771)	0.702 (0.673-0.730)	0.723 (0.694-0.752)	0.721 (0.691-0.749)	0.721 (0.690-0.752)
LLM2	0.736 (0.704-0.770)	0.808 (0.784-0.831)	0.736 (0.706-0.766)	0.720 (0.691-0.750)	0.712 (0.683-0.743)	0.710 (0.679-0.741)
LLM3	0.700 (0.667-0.734)	0.776 (0.750-0.801)	0.697 (0.666-0.728)	0.704 (0.675-0.735)	0.700 (0.671-0.731)	0.700 (0.670-0.731)
Text-embedding 1						
SVM	0.736 (0.646-0.818)	0.770 (0.708-0.822)	0.686 (0.612-0.754)	0.711 (0.638-0.784)	0.678 (0.610-0.746)	0.675 (0.605-0.745)
LR	0.758 (0.671-0.842)	0.793 (0.737-0.840)	0.727 (0.657, 0787)	0.715 (0.625-0.787)	0.650 (0.582-0.723)	0.647 (0.576-0.717)
XGB	0.841 (0.783-0.897)	0.822 (0.770-0.869)	0.737 (0.663-0.804)	0.724 (0.650-0.795)	0.672 (0.605-0.746)	0.662 (0.591-0.732)
NN	0.802 (0.725-0.878)	0.817 (0.775-0.864)	0.736 (0.668-0.806)	0.739 (0.665-0.807)	0.661 (0.588-0.723)	0.656 (0.582-0.720)
Text-embedding 2						
SVM	0.626 (0.532-0.719)	0.685 (0.624-0.746)	0.592 (0.522-0.660)	0.633 (0.549-0.713)	0.548 (0.475-0.621)	0.546 (0.468-0.621)
LR	0.642 (0.548-0.733)	0.704 (0.648-0.761)	0.621 (0.551-0.689)	0.637 (0.556-0.716)	0.571 (0.497-0.644)	0.569 (0.494-0.644)
XGB	0.747 (0.672-0.822)	0.775 (0.718-0.831)	0.607 (0.526-0.684)	0.681 (0.604-0.755)	0.667 (0.599-0.729)	0.660 (0.587-0.727)
NN	0.699 (0.616-0.779)	0.728 (0.662-0.779)	0.631 (0.556-0.698)	0.695 (0.611-0.766)	0.616 (0.548-0.684)	0.600 (0.532-0.669)
Text-embedding 3						
SVM	0.641 (0.551-0.728)	0.700 (0.638-0.765)	0.608 (0.536-0.673)	0.619 (0.536-0.700)	0.588 (0.514-0.655)	0.585 (0.511-0.654)
LR	0.657 (0.565-0.745)	0.723 (0.662-0.779)	0.636 (0.560-0.702)	0.636 (0.554-0.717)	0.588 (0.514-0.655)	0.585 (0.508-0.654)
XGB	0.755 (0.680-0.821)	0.756 (0.695-0.812)	0.565 (0.488-0.637)	0.736 (0.661-0.804)	0.718 (0.655-0.780)	0.713 (0.648-0.778)
NN	0.721 (0.642-0.803)	0.765 (0.704-0.817)	0.648 (0.563-0.718)	0.701 (0.623-0.780)	0.621 (0.548-0.695)	0.620 (0.546-0.694)

Abbreviations: AUROC, area under the receiver operating characteristic curve; LLM1, GPT-4o (May 13, 2024, version; OpenAI); LLM2, gemini-1.0-pro (February 2024 version; Google); LLM3, GPT-3.5-turbo-16k (January 25, 2024, version; OpenAI); LR, logistic regression; NN, neural network; SCT, sentence completion test; SVM, support vector machine; text-embedding 1, text-embedding-3-large (OpenAI), text-embedding 2, text-embedding-3-small (OpenAI), and text-embedding 3, text-embedding-002-ada (OpenAI); XGB, extreme gradient boosting.

### Depression Detection

Self-concept narratives consistently showed the most effective results for all LLMs across both zero-shot and few-shot learning (Table 3; eTables 2-4 in Supplement 1). In zero-shot learning, LLM1 (AUROC, 0.720 [95% CI, 0.689-0.752]), LLM2 (AUROC, 0.714 [95% CI, 0.680-0.747]), and LLM3 (AUROC, 0.731 [95% CI, 0.703-0.761]) showed comparable performance (Table 3), with minimal mean AUROC differences between models (eTable 5 in Supplement 1). Few-shot learning was significantly associated with improved model performance, with LLM1 showing the best discriminant ability among LLMs, achieving an AUROC of 0.754 (95% CI, 0.721-0.781); LLM2 had an AUROC of 0.736 (95% CI, 0.704-0.770) and LLM 3 had an AUROC of 0.700 (95% CI, 0.667-0.734). Statistical analysis using the Cochran *Q* test (*Q* = 159.6; *P* < .001) revealed a significant difference in accuracy among models (eTable 6 in Supplement 1). We found that LLM3 underperformed compared with LLM1 and LLM2 in zero-shot learning, with the McNemar test indicating significant differences (LLM1 vs LLM3: χ^2^ = 64.89; *P* < .001; and LLM2 vs LLM3: χ^2^ = 62.95; *P* < .001) (eTable 7 in Supplement 1).

Among embedding-based ML models that were also based on self-concept narratives, text-embedding 1 with XGB achieved the highest AUROC of 0.841 (95% CI, 0.783-0.897), an accuracy of 0.822 (95% CI, 0.770-0.869), and a macro F1-score of 0.737 (95% CI, 0.663-0.804) (Table 3). NN and LR also performed strongly with text-embedding 1 model, showing AUROCs of 0.802 (95% CI, 0.725-0.878) and 0.758 (95% CI, 0.671-0.842), respectively, with accuracies and macro F1-scores above 0.7. The larger embedding model, text-embedding 1, showed statistically superior performance compared with smaller models. The text-embedding 1 model with XGB outperformed text-embedding 2 with SVM, LR, or NN, achieving an AUROC of 0.841 (95% CI, 0.783-0.897) for depression, as well as text-embedding 3 with SVM or LR (eTable 8 in Supplement 1). Although text-embedding 2 and text-embedding 3 showed lower performance, they maintained notable effectiveness, with XGB achieving AUROCs of 0.747 (95% CI, 0.672-0.822) and 0.755 (95% CI, 0.680-0.821), respectively.

### Suicide Risk Detection

Suicide risk detection was more challenging overall, with lower performance compared with depression detection (Table 3; eTables 2-4 in Supplement 1). For self-concept narratives, zero-shot LLM1 achieved the highest AUROC of 0.731 (95% CI, 0.704-0.762), and LLM2 performed comparably with an AUROC of 0.721 (95% CI, 0.695-0.750). Zero-shot LLM3 significantly underperformed zero-shot LLM1 and LLM2, with mean AUROC differences of 0.0945 (95% CI, 0.0636-0.1231) and 0.0843 (95% CI, 0.0510-0.1174), respectively (eTable 5 in Supplement 1). LLM1 showed significantly higher accuracy than LLM3 (χ^2^ = 20.35; *P* < .001), while LLM2 did not after Bonferroni correction (χ^2^ = 8.461; *P* = .004; threshold, *P* < .003) (eTable 7 in Supplement 1). Few-shot learning improved performance, particularly for LLM3 (mean AUROC difference of 0.0690 [95% CI, 0.0359-0.1012]) (eTable 5 in Supplement 1). However, zero-shot and few-shot performances were comparable within each model, with mean AUROC differences of 0.0068 (95% CI, −0.0183 to 0.0338) for LLM1 and −0.001 (95% CI, −0.0207 to 0.0187]) for LLM2.

Based on self-concept narratives, embedding-based ML models maintained reasonable accuracy, although the performance was lower than in depression detection. Text-embedding 1 with NN achieved the highest AUROC of 0.739 (95% CI, 0.665-0.807), an accuracy of 0.661 (95% CI, 0.588-0.723), and a macro F1-score of 0.656 (95% CI, 0.582-0.720), closely followed by XGB (AUROC, 0.724 [95% CI, 0.650-0.795]) (Table 3). Performance gaps between different embedding sizes were smaller compared with depression detection, with more consistent performance across ML models (AUROC range, 0.711 [95% CI, 0.638-0.784] to 0.739 [0.665-0.807] for text-embedding 1). Text-embedding 3 with XGB showed competitive performance (AUROC, 0.736 [95% CI, 0.661-0.804]), while text-embedding 2 with XGB maintained moderate performance (AUROC, 0.681 [95% CI, 0.604-0.755]).

### Qualitative Analysis

The patient in case 1 was clinically significantly identified as having depression by LLM1 based on self-concept narratives, but not as having depression based on gender perception narratives (eTable 9 in Supplement 1). The self-concept narratives contained negative self-image and pessimistic thoughts about the future, while the gender perception narratives showed no signs of distorted beliefs about gender roles (eResults in Supplement 1). For cases 2 and 3, in which both patients had clinically significant depression, LLM1 failed to detect depression based on self-concept narratives (eTable 9 in Supplement 1). The patient in case 2 had a defensive response style, attempting to present a positive self-image, and the patient in case 3 provided rather superficial responses (eResults in Supplement 1).

## Discussion

The purpose of this study was to assess whether LLMs and text-embedding models can identify clinically significant depression and high risk of suicide based on patients’ narratives collected from the SCT. We found that both LLMs and embedding-based ML models successfully detected depression and suicide, achieving AUROCs of approximately 0.7. This finding suggests that domain-specific pretraining may not be essential, as general-purpose, non–domain-specific LLMs can sufficiently interpret the sentiment in patients’ narratives. Specifically, both zero-shot and few-shot LLM1 and LLM2 showed the highest AUROC among the LLMs, along with ML models trained on embeddings from the text-embedding 1 model. Self-concept narratives yielded the highest performance, probably because they might effectively reflect the cognitive patterns of patients with depression and suicide risk. These findings underline the potential of both advanced generative LLMs and ML models using high-quality embeddings for mental health prediction tasks in clinical settings.

Our models showed performance comparable to previous studies using social media text data by zero-shot learning to detect stress or depression.^12,14,15,17,44^ Also, compared with the study by Bartal et al,^19^ which showed limited performance of LLM3 in detecting childbirth-related posttraumatic stress disorder, our study showed better results, likely due to the use of the latest generative LLMs and embedding models. This finding is in line with our results that models trained on larger datasets performed better, with LLM1, LLM2, and the text-embedding 1 model showing the greatest performance. Although the exact parameters used for LLM1 were not disclosed, it is estimated that LLM1 was trained on 200 billion parameters, and the text-embedding 1 model might have been similarly trained on an extensive dataset of comparable scale. Embedding-based ML models showed the best performance, likely because they were specifically tailored to participants from our institution.

However, even LLM3 showed high performance in our zero-shot and few-shot learning, suggesting that either detecting depression-related risk is more suitable for language models or SCT narratives effectively reflect the psychological state of participants at risk. The self-concept narratives, revealing attitudes toward one’s abilities, guilt, goals, past, and future,^22^ align with the depression triad of Beck.^20^ These characteristics of narratives may provide more insights into the presence of depression, possibly explaining the higher discriminative power observed in the present study. The importance of narrative content is further supported by lower performance with other types of narratives, especially gender perception narratives, and confirmed by our qualitative analyses. Although standard self-assessment scales are useful, time efficient, and easier to administer at the screening stage, individuals who may otherwise be defensive could reveal their depressive cognitions more openly through projective tests such as the SCT.^21,22,26^ These findings call for a reevaluation of the previously undervalued significance of SCT, particularly by leveraging LLMs to analyze these narratives, potentially improving the effectiveness of screening for mental health risks.

Through qualitative analyses, we identified factors associated with LLM performance beyond the narrative content. Defensive or superficial responses, often due to psychological conflict or low motivation, may limit detection of mental health risks.^45^ These findings suggest that, like all psychological assessments, SCT narratives alone cannot definitively determine mental health risks. Clinical interviews, comprehensive psychiatric history taking, and clinical observations remain essential.

To our knowledge, this is the first study to examine the use of LLMs and text-embedding models for detecting depression and suicide risk based on semistructured narratives of psychiatric patients. Our study was based on data from Korean-speaking psychiatric patients. Although LLMs are trained in multiple languages, including Korean, and can translate effectively, our results were comparable with those from studies based on English data.^12,17,44^ This finding suggests that LLMs have advanced to the point where they can make accurate predictions across different languages, contributing to a broader understanding of mental health prediction across diverse linguistic contexts.

### Limitations

This study has several limitations. First, this study was based on narrative data of patients who visited a psychiatric clinic. Even those not classified in the clinically significant depression group or high risk of suicide group might have had other psychiatric symptoms, such as anxiety, mild depression, or psychotic symptoms, limiting generalizability. External validation with data from other psychiatric institutions or a nonpsychiatric population would be necessary. Second, depression and suicide severity were determined using self-report measures rather than clinical diagnoses, so individuals with other psychiatric conditions experiencing significant depressive symptoms or suicidal ideation might have been included. This suggests the model may detect general psychological distress rather than specific conditions, limiting its diagnostic capability. Third, the dataset for depression classification was imbalanced, which could potentially affect model performance. However, we primarily compared AUROCs between models, and the AUROC values were consistent with balanced accuracy for LLM evaluation, indicating that model performance was well balanced despite data imbalances. Fourth, although we performed qualitative analysis to identify factors associated with model prediction, the specific narrative features influencing prediction remain unclear due to the “black-box” nature of AI models, whose internal works are not transparent.^46^ Future studies using explainable AI could clarify which features are most diagnostically significant for detecting mental health risks.^46^ Fifth, although we used deidentified narratives, deploying LLMs in clinical practice raises ethical concerns that must be carefully addressed in the future.

## Conclusions

In this cross-sectional study of SCT narratives from psychiatric patients, LLMs and text-embedding models effectively detected depression and suicide risk, particularly using self-concept narratives. Although these models demonstrate potential for detecting mental health risks, further improvements in performance and safety are essential before clinical application.

## Notes

Supplement 1. **eTable 1.** Translated Version of 50 Items of the Korean Sentence Completion Test **eTable 2.** Performance of Zero-Shot, Few-Shot Large Language Models and Embedding-Based Machine Learning Models Detecting Clinically Significant Depression and High Risk of Suicide Based on SCT Family Narratives **eTable 3.** Performance of Zero-Shot, Few-Shot Large Language Models and Embedding-Based Machine Learning Models Detecting Clinically Significant Depression and High Risk of Suicide Based on SCT Gender Perception Narratives **eTable 4.** Performance of Zero-Shot, Few-Shot Large Language Models and Embedding-Based Machine Learning Models Detecting Clinically Significant Depression and High Risk of Suicide Based on SCT Interpersonal Relations **eTable 5.** Mean AUC Differences and 95% Confidence Intervals Between Model Pairs for Large Language Models Using Self-Concept Narratives **eTable 6.** Cochran’s Test Results for Large Language Models Accuracy Comparisons Using Self-Concept Narratives **eTable 7.** McNemar Test Results for Pairwise Model Comparisons Using Self-Concept Narratives **eTable 8.** Statistically Significant Mean AUROC Differences and 95% Confidence Intervals Between Model Pairs for Embedding-Based ML Models Using Self-Concept Narratives **eMethods.** Procedures for Qualitative Analysis **eTable 9.** Narratives and GPT-4o Reasoning in Qualitative Analysis **eResults.** Case Summaries of Qualitative Analysis

Supplement 2.
Data Sharing Statement
