# Supplementary material for: Large Language Models and Text Embeddings for Detecting Depression and Suicide in Patient Narratives
Source: JAMA Netw Open. 2025 May 23;8(5):e2511922. doi: 10.1001/jamanetworkopen.2025.11922 (PMC12102709; doi:10.1001/jamanetworkopen.2025.11922)
Supplement: Supplement 1. — eTable 1. Translated Version of 50 Items of the Korean Sentence Completion Test eTable 2. Performance of Zero-Shot, Few-Shot Large Language Models and Embedding-Based Machine Learning Models Detecting Clinically Significant Depression and High Risk of Suicide Based on SCT Family Narratives eTable 3. Performance of Zero-Shot, Few-Shot Large Language Models and Embedding-Based Machine Learning Models Detecting Clinically Significant Depression and High Risk of Suicide Based on SCT Gender Perception Narratives eTable 4. Performance of Zero-Shot, Few-Shot Large Language Models and Embedding-Based Machine Learning Models Detecting Clinically Significant Depression and High Risk of Suicide Based on SCT Interpersonal Relations eTable 5. Mean AUC Differences and 95% Confidence Intervals Between Model Pairs for Large Language Models Using Self-Concept Narratives eTable 6. Cochran’s Test Results for Large Language Models Accuracy Comparisons Using Self-Concept Narratives eTable 7. McNemar Test Results for Pairwise Model Comparisons Using Self-Concept Narratives eTable 8. Statistically Significant Mean AUROC Differences and 95% Confidence Intervals Between Model Pairs for Embedding-Based ML Models Using Self-Concept Narratives eMethods. Procedures for Qualitative Analysis eTable 9. Narratives and GPT-4o Reasoning in Qualitative Analysis eResults. Case Summaries of Qualitative Analysis [file jamanetwopen-e2511922-s001.pdf]

Supplementary Online Content

Lho SK, Park SC, Lee H, et al. Large language models and text embeddings for detecting depression and suicide in patient narratives. *JAMA Netw Open.* 2025;8(5):e2511922. doi:10.1001/jamanetworkopen.2025.11922

eTable 1. Translated Version of 50 Items of the Korean Sentence Completion Test
eTable 2. Performance of Zero-Shot, Few-Shot Large Language Models and Embedding-Based Machine Learning Models Detecting Clinically Significant Depression and High Risk of Suicide Based on SCT Family Narratives
eTable 3. Performance of Zero-Shot, Few-Shot Large Language Models and Embedding-Based Machine Learning Models Detecting Clinically Significant Depression and High Risk of Suicide Based on SCT Gender Perception Narratives
eTable 4. Performance of Zero-Shot, Few-Shot Large Language Models and Embedding-Based Machine Learning Models Detecting Clinically Significant Depression and High Risk of Suicide Based on SCT Interpersonal Relations
eTable 5. Mean AUROC Differences and 95% Confidence Intervals Between Model Pairs for Large Language Models Using Self-Concept Narratives
eTable 6. Cochran's Test Results for Large Language Models Accuracy Comparisons Using Self-Concept Narratives
eTable 7. McNemar Test Results for Pairwise Model Comparisons Using Self-Concept Narratives
eTable 8. Statistically Significant Mean AUROC Differences and 95% Confidence Intervals Between Model Pairs for Embedding-Based ML Models Using Self-Concept Narratives
eMethods. Procedures for Qualitative Analysis
eTable 9. Narratives and GPT-4o Reasoning in Qualitative Analysis
eResults. Case Summaries of Qualitative Analysis

This supplementary material has been provided by the authors to give readers additional information about their work.

© 2025 Lho SK et al. *JAMA Network Open.*

eTable 1. Translated Version of 50 Items of the Korean Sentence Completion Test

Item	Sentence	Category
1	Supporting a family is	Family
2	Someday I will	Self-concept
3	My mother is	Family
4	When people avoid me	Interpersonal relations
5	Education is	Interpersonal relations
6	When people talk about sex	Gender perception
7	To be truly happy, I need to	Self-concept
8	I am	Self-concept
9	Being with other people is	Interpersonal relations
10	The kind of woman I desire is	Gender perception
11	When I see a pregnant woman	Gender perception
12	Children dislike group activities because	Interpersonal relations
13	My wife (or husband) is	Gender perception
14	My good points are	Self-concept
15	The ideal man is	Gender perception
16	The people I work with are usually	Interpersonal relations
17	My father is	Family
18	When I get irritated	Self-concept
19	My father and I are	Family
20	Compared to other families, my family is	Family
21	Women are lucky because	Gender perception
22	When I’m not around, my friends	Interpersonal relations
23	I think men are	Gender perception
24	My mother and I are	Family
25	What I want to forget at all costs is	Self-concept
26	I think sometimes my father is	Family
27	Men are lucky because	Gender perception
28	My biggest problem is	Self-concept
29	For a woman to have a career is	Gender perception
30	What bothers me is	Self-concept
31	What I want most is	Self-concept
32	About married life, I	Gender perception
33	When I was young	Self-concept
34	What I dislike most about men is	Gender perception
35	I feel guilty about	Self-concept
36	If I could be young again, I would	Self-concept
37	A good mother is	Family
38	Most fathers are	Family
39	I think women are	Gender perception
40	When things don’t go as planned	Self-concept
41	I think a true friend is	Interpersonal relations
42	When I see a superior coming, I	Interpersonal relations
43	When my mother hits me	Family
44	When something strange happens to me	Self-concept
45	When I get old	Self-concept
46	The biggest disadvantage of being a woman is	Gender perception
47	Superiors are	Interpersonal relations
48	When I’m with friends of the opposite sex	Gender perception
49	Men think about women	Gender perception

© 2025 Lho SK et al. *JAMA Network Open.*

50	My abilities are	Self-concept

© 2025 Lho SK et al. *JAMA Network Open.*

eTable 2. Performance of Zero-Shot, Few-Shot Large Language Models and Embedding-Based Machine Learning Models Detecting Clinically Significant Depression and High Risk of Suicide Based on SCT Family Narratives

Category and model	Depression			Suicide		
	AUROC (95% CI)	Accuracy (95% CI)	Macro F1 (95% CI)	AUROC (95% CI)	Accuracy (95% CI)	Macro F1 (95% CI)
Zero-shot						
LLM1	0.651 (0.615-0.684)	0.662 (0.633-0.688)	0.611 (0.581-0.645)	0.601 (0.577-0.624)	0.621 (0.588-0.653)	0.569 (0.538-0.600)
LLM2	0.653 (0.621-0.687)	0.659 (0.633-0.688)	0.610 (0.579-0.640)	0.617 (0.588-0.643)	0.633 (0.601-0.666)	0.601 (0.567-0.633)
LLM3	0.621 (0.590-0.655)	0.584 (0.555-0.613)	0.555 (0.524-0.586)	0.594 (0.569-0.617)	0.613 (0.583-0.646)	0.565 (0.529-0.597)
Few-shot						
LLM1	0.640 (0.608-0.674)	0.625 (0.599-0.654)	0.586 (0.555-0.615)	0.613 (0.587-0.639)	0.631 (0.600-0.665)	0.592 (0.558-0.625)
LLM2	0.647 (0.614-0.681)	0.674 (0.645-0.703)	0.615 (0.584-0.644)	0.624 (0.591-0.656)	0.620 (0.590-0.653)	0.619 (0.588-0.651)
LLM3	0.660 (0.623-0.693)	0.688 (0.661-0.716)	0.629 (0.596-0.659)	0.648 (0.617-0.679)	0.657 (0.626-0.689)	0.645 (0.615-0.677)
Text-embedding 1						
SVM	0.579 (0.499-0.659)	0.624 (0.563-0.686)	0.491 (0.427-0.558)	0.598 (0.515-0.679)	0.559 (0.479-0.626)	0.554 (0.479-0.626)
LR	0.585 (0.504-0.661)	0.620 (0.559-0.685)	0.488 (0.426-0.555)	0.600 (0.515-0.678)	0.582 (0.508-0.655)	0.577 (0.503-0.654)
XGB	0.659 (0.572-0.739)	0.751 (0.690-0.808)	0.493 (0.430-0.559)	0.663 (0.579-0.739)	0.627 (0.559-0.695)	0.618 (0.546-0.688)
NN	0.620 (0.538-0.710)	0.685 (0.624-0.742)	0.539 (0.465-0.611)	0.674 (0.595-0.752)	0.638 (0.565-0.706)	0.636 (0.564-0.706)
Text-embedding 2						
SVM	0.603 (0.520-0.691)	0.662 (0.596-0.723)	0.569 (0.498-0.641)	0.553 (0.465-0.645)	0.559 (0.486-0.633)	0.553 (0.477-0.630)
LR	0.596 (0.506-0.684)	0.662 (0.596-0.723)	0.564 (0.489-0.636)	0.557 (0.468-0.649)	0.559 (0.486-0.633)	0.552 (0.473-0.627)
XGB	0.619 (0.533-0.696)	0.737 (0.681-0.793)	0.471 (0.420-0.530)	0.619 (0.533-0.698)	0.599 (0.525-0.672)	0.589 (0.511-0.662)
NN	0.615 (0.530-0.695)	0.718 (0.657-0.779)	0.563 (0.490-0.641)	0.594 (0.507-0.683)	0.565 (0.486-0.638)	0.550 (0.470-0.624)
Text-embedding 3						
SVM	0.547 (0.453-0.643)	0.643 (0.577-0.709)	0.503 (0.438-0.577)	0.555 (0.471-0.637)	0.548 (0.480-0.621)	0.548 (0.478-0.621)
LR	0.544 (0.447-0.640)	0.667 (0.601-0.732)	0.539 (0.466-0.613)	0.558 (0.475-0.639)	0.542(0.475-0.610)	0.542 (0.472-0.610)
XGB	0.720 (0.644-0.790)	0.765 (0.700-0.822)	0.540 (0.463-0.615)	0.603 (0.512-0.682)	0.559 (0.480-0.633)	0.556 (0.476-0.626)
NN	0.583 (0.490-0.670)	0.690 (0.629-0.746)	0.511 (0.446-0.574)	0.610 (0.524-0.691)	0.565 (0.486-0.644)	0.559 (0.480-0.691)

Abbreviations: AUROC, area under the receiver operating curve; CI, confidence interval; LLM1, GPT-4o (May 13, 2024, version; OpenAI); LLM2, gemini-1.0-pro (February 2024 version; Google); LLM3, GPT-3.5-turbo-16k (January 25, 2024, version; OpenAI); LR, logistic regression; NN, neural network; SCT, sentence completion test; SVM, Support vector machine; text-embedding 1, text-embedding-3-large (OpenAI); text-embedding 2, text-embedding-3-small (OpenAI); text-embedding 3, text-embedding-002-ada (OpenAI); XGB, extreme gradient boosting.

© 2025 Lho SK et al. *JAMA Network Open.*

eTable 3. Performance of Zero-Shot, Few-Shot Large Language Models and Embedding-Based Machine Learning Models Detecting Clinically Significant Depression and High Risk of Suicide Based on SCT Gender Perception Narratives

Category and model	Depression			Suicide		
	AUROC (95% CI)	Accuracy (95% CI)	Macro F1 (95% CI)	AUROC (95% CI)	Accuracy (95% CI)	Macro F1 (95% CI)
Zero-shot						
LLM1	0.627 (0.593-0.659)	0.582 (0.553-0.612)	0.556 (0.523-0.590)	0.561 (0.543-0.580)	0.588 (0.556-0.624)	0.480 (0.447-0.512)
LLM2	0.625 (0.593-0.658)	0.571 (0.542-0.602)	0.549 (0.520-0.580)	0.533 (0.518-0.548)	0.561 (0.529-0.596)	0.436 (0.409-0.465)
LLM3	0.542 (0.520-0.560)	0.341 (0.314-0.368)	0.334 (0.307-0.360)	0.500 (0.497-0.504)	0.533 (0.501-0.568)	0.350 (0.335-0.365)
Few-shot						
LLM1	0.610 (0.583-0.633)	0.463 (0.436-0.492)	0.462 (0.433-0.493)	0.602 (0.576-0.626)	0.621 (0.589-0.653)	0.572 (0.538-0.605)
LLM2	0.628 (0.593-0.661)	0.621 (0.591-0.649)	0.579 (0.550-0.608)	0.582 (0.555-0.608)	0.600 (0.566-0.633)	0.552 (0.520-0.583)
LLM3	0.586 (0.548-0.619)	0.555 (0.525-0.583)	0.526 (0.496-0.556)	0.581 (0.550-0.611)	0.589 (0.557-0.622)	0.578 (0.543-0.612)
Text-embedding 1						
SVM	0.607 (0.518-0.701)	0.681 (0.620-0.742)	0.562 (0.491-0.631)	0.634 (0.552-0.713)	0.571 (0.497-0.644)	0.555 (0.477-0.629)
LR	0.627 (0.540-0.719)	0.695 (0.634-0.756)	0.584 (0.511-0.653)	0.641 (0.559-0.723)	0.576 (0.503-0.650)	0.564 (0.487-0.636)
XGB	0.650 (0.568-0.735)	0.723 (0.662-0.784)	0.436 (0.402-0.479)	0.590 (0.507-0.670)	0.565 (0.486-0.633)	0.550 (0.473-0.620)
NN	0.649 (0.563-0.736)	0.732 (0.671-0.789)	0.581 (0.505-0.655)	0.622 (0.545-0.705)	0.582 (0.508-0.655)	0.557 (0.503-0.648)
Text-embedding 2						
SVM	0.672 (0.579-0.758)	0.714 (0.653-0.775)	0.615 (0.539-0.686)	0.647 (0.565-0.729)	0.621 (0.548-0.695)	0.612 (0.539-0.685)
LR	0.683 (0.592-0.771)	0.709 (0.643-0.770)	0.615 (0.536-0.686)	0.645 (0.560-0.728)	0.650 (0.576-0.723)	0.641 (0.564-0.713)
XGB	0.635 (0.547-0.716)	0.746 (0.685-0.803)	0.476 (0.421-0.537)	0.617 (0.524-0.699)	0.610 (0.537-0.684)	0.605 (0.530-0.675)
NN	0.572 (0.477-0.660)	0.695 (0.629-0.756)	0.546 (0.471-0.617)	0.595 (0.500-0.678)	0.559 (0.480-0.633)	0.545 (0.468-0.616)
Text-embedding 3						
SVM	0.593 (0.504-0.684)	0.685 (0.620-0.746)	0.571 (0.497-0.640)	0.610 (0.524-0.695)	0.605 (0.525-0.678)	0.600 (0.522-0.672)
LR	0.601 (0.510-0.692)	0.667 (0.601-0.723)	0.545 (0.472-0.612)	0.609 (0.523-0.691)	0.593 (0.508-0.661)	0.588 (0.503-0.657)
XGB	0.748 (0.675-0.817)	0.751 (0.695-0.808)	0.506 (0.439-0.573)	0.605 (0.515-0.688)	0.559 (0.480-0.633)	0.543 (0.466-0.620)
NN	0.684 (0.599-0.757)	0.737 (0.676-0.793)	0.532 (0.457-0.604)	0.616 (0.531-0.698)	0.559 (0.486-0.633)	0.549 (0.475-0.623)

Abbreviations: AUROC, area under the receiver operating curve; CI, confidence interval; LLM1, GPT-4o (May 13, 2024, version; OpenAI); LLM2, gemini-1.0-pro (February 2024 version; Google); LLM3, GPT-3.5-turbo-16k (January 25, 2024, version; OpenAI); LR, logistic regression; NN, neural network; SCT, sentence completion test; SVM, Support vector machine; text-embedding 1, text-embedding-3-large (OpenAI); text-embedding 2, text-embedding-3-small (OpenAI); text-embedding 3, text-embedding-002-ada (OpenAI); XGB, extreme gradient boosting.

© 2025 Lho SK et al. *JAMA Network Open.*

eTable 4. Performance of Zero-Shot, Few-Shot Large Language Models and Embedding-Based Machine Learning Models Detecting Clinically Significant Depression and High Risk of Suicide Based on SCT Interpersonal Relations

Category and model	Depression			Suicide		
	AUROC (95% CI)	Accuracy (95% CI)	Macro F1 (95% CI)	AUROC (95% CI)	Accuracy (95% CI)	Macro F1 (95% CI)
Zero-shot						
LLM1	0.665 (0.634-0.696)	0.768 (0.742-0.791)	0.670 (0.638-0.700)	0.583 (0.557-0.610)	0.603 (0.569-0.634)	0.549 (0.513-0.584)
LLM2	0.677 (0.646-0.707)	0.650 (0.621-0.677)	0.615 (0.584-0.645)	0.575 (0.552-0.599)	0.598 (0.565-0.631)	0.531 (0.497-0.563)
LLM3	0.635 (0.598-0.666)	0.689 (0.660-0.716)	0.616 (0.583-0.647)	0.521 (0.512-0.530)	0.552 (0.519-0.587)	0.392 (0.369-0.416)
Few-shot						
LLM1	0.669 (0.634-0.701)	0.682 (0.654-0.709)	0.630 (0.599-0.659)	0.619 (0.588-0.649)	0.620 (0.588-0.649)	0.619 (0.588-0.649)
LLM2	0.658 (0.626-0.690)	0.759 (0.733-0.787)	0.661 (0.626-0.695)	0.618 (0.589-0.645)	0.605 (0.573-0.638)	0.594 (0.562-0.627)
LLM3	0.638 (0.606-0.673)	0.737 (0.710-0.763)	0.637 (0.603-0.670)	0.603 (0.571-0.634)	0.609 (0.580-0.642)	0.601 (0.570-0.631)
Text-embedding 1						
SVM	0.673 (0.592-0.749)	0.690 (0.629-0.751)	0.609 (0.538-0.676)	0.640 (0.555-0.719)	0.588 (0.508-0.661)	0.586 (0.507-0.657)
LR	0.682 (0.599-0.759)	0.700 (0.638-0.761)	0.625 (0.555-0.694)	0.653 (0.568-0.729)	0.593 (0.520-0.667)	0.591 (0.518-0.662)
XGB	0.720 (0.645-0.787)	0.723 (0.662-0.779)	0.523 (0.453-0.589)	0.725 (0.646-0.793)	0.650 (0.576-0.712)	0.645 (0.570-0.709)
NN	0.706 (0.626-0.782)	0.732 (0.676-0.793)	0.644 (0.570-0.715)	0.628 (0.544-0.709)	0.582 (0.508-0.655)	0.575 (0.499-0.643)
Text-embedding 2						
SVM	0.635 (0.537-0.728)	0.709 (0.643-0.765)	0.577 (0.503-0.650)	0.616 (0.529-0.703)	0.616 (0.542-0.689)	0.616 (0.529-0.703)
LR	0.640 (0.545-0.735)	0.709 (0.643-0.770)	0.583 (0.509-0.655)	0.628 (0.545-0.709)	0.599 (0.531-0.667)	0.597 (0.529-0.666)
XGB	0.710 (0.634-0.790)	0.784 (0.728-0.836)	0.588 (0.509-0.674)	0.662 (0.579-0.737)	0.655 (0.588-0.723)	0.644 (0.572-0.714)
NN	0.646 (0.562-0.735)	0.732 (0.676-0.789)	0.588 (0.513-0.664)	0.664 (0.585-0.743)	0.610 (0.542-0.678)	0.604 (0.534-0.677)
Text-embedding 3						
SVM	0.556 (0.473-0.644)	0.657 (0.596-0.723)	0.550 (0.480-0.621)	0.631 (0.550-0.713)	0.582 (0.508-0.655)	0.579 (0.507-0.649)
LR	0.588 (0.505-0.674)	0.657 (0.592-0.723)	0.550 (0.481-0.621)	0.630 (0.549-0.715)	0.593 (0.520-0.667)	0.589 (0.517-0.660)
XGB	0.712 (0.638-0.780)	0.704 (0.643-0.765)	0.479 (0.417-0.548)	0.631 (0.547-0.714)	0.633 (0.559-0.706)	0.627 (0.550-0.698)
NN	0.706 (0.634-0.774)	0.695 (0.634-0.756)	0.584 (0.634-0.774)	0.610 (0.526-0.690)	0.582 (0.508-0.655)	0.578 (0.507-0.653)

Abbreviations: AUROC, area under the receiver operating curve; CI, confidence interval; LLM1, GPT-4o (May 13, 2024, version; OpenAI); LLM2, gemini-1.0-pro (February 2024 version; Google); LLM3, GPT-3.5-turbo-16k (January 25, 2024, version; OpenAI); LR, logistic regression; NN, neural network; SCT, sentence completion test; SVM, Support vector machine; text-embedding 1, text-embedding-3-large (OpenAI); text-embedding 2, text-embedding-3-small (OpenAI); text-embedding 3, text-embedding-002-ada (OpenAI); XGB, extreme gradient boosting.

© 2025 Lho SK et al. *JAMA Network Open.*

eTable 5. Mean AUROC Differences and 95% Confidence Intervals Between Model Pairs for Large Language Models Using Self-Concept Narratives

Depression detection	Mean AUROC difference[a]	95% CI lower	95% CI upper
ZS LLM1 vs. ZS LLM2	0.0045	-0.0231	0.0317
ZS LLM1 vs. ZS LLM3	-0.0125	-0.0479	0.0214
ZS LLM1 vs. FS LLM1	-0.0362	-0.0669	-0.006
ZS LLM1 vs. FS LLM2	-0.0184	-0.0436	0.0052
ZS LLM1 vs. FS LLM3	0.0164	-0.0124	0.0465
ZS LLM2 vs. ZS LLM3	-0.017	-0.0528	0.0199
ZS LLM2 vs. FS LLM1	-0.0408	-0.0732	-0.0049
ZS LLM2 vs. FS LLM2	-0.0229	-0.0471	0.0021
ZS LLM2 vs. FS LLM3	0.0119	-0.0199	0.0458
ZS LLM3 vs. FS LLM1	-0.0237	-0.0582	0.0096
ZS LLM3 vs. FS LLM2	-0.0059	-0.0402	0.0301
ZS LLM3 vs. FS LLM3	0.0289	-0.0045	0.0666
FS LLM1 vs. FS LLM2	0.0178	-0.0104	0.047
FS LLM1 vs. FS LLM3	0.0527	0.0209	0.0868
FS LLM2 vs. FS LLM3	0.0349	0.0059	0.0654
**Suicide risk detection**	**Mean AUROC difference[a]**	**95% CI lower**	**95% CI upper**
ZS LLM1 vs. ZS LLM2	0.0102	-0.0177	0.0358
ZS LLM1 vs. ZS LLM3	0.0945	0.0636	0.1241
ZS LLM1 vs. FS LLM1	0.0068	-0.0183	0.0338
ZS LLM1 vs. FS LLM2	0.0093	-0.0166	0.0347
ZS LLM1 vs. FS LLM3	0.0255	-0.0026	0.0538
ZS LLM2 vs. ZS LLM3	0.0843	0.0510	0.1174
ZS LLM2 vs. FS LLM1	-0.0034	-0.0293	0.0229
ZS LLM2 vs. FS LLM2	-0.001	-0.0207	0.0187
ZS LLM2 vs. FS LLM3	0.0153	-0.0127	0.0448
ZS LLM3 vs. FS LLM1	-0.0877	-0.1209	-0.0562
ZS LLM3 vs. FS LLM2	-0.0852	-0.1177	-0.0515
ZS LLM3 vs. FS LLM3	0.0690	0.0359	0.1012
FS LLM1 vs. FS LLM2	0.0025	-0.0218	0.0271
FS LLM1 vs. FS LLM3	0.0187	-0.0089	0.0461
FS LLM2 vs. FS LLM3	0.0162	-0.0107	0.0433

Abbreviations: AUROC, area under the receiver operating curve; CI, confidence interval; FS, few-shot; LLM1, GPT-4o (May 13, 2024, version; OpenAI); LLM2, gemini-1.0-pro (February 2024 version; Google; LLM3), GPT-3.5-turbo-16k (January 25, 2024, version; OpenAI); ZS, zero-shot.

[a]The mean AUROC difference is considered statistically significant when the 95% confidence interval does not include zero.

© 2025 Lho SK et al. *JAMA Network Open.*

eTable 6. Cochran's Test Results for Large Language Models Accuracy Comparisons Using Self-Concept Narratives

Task type	N	Cochran' Q	df	*p* value
Depression detection	1,060	159.6	5	<.001
Suicide risk detection	878	29.1	5	<.001

© 2025 Lho SK et al. *JAMA Network Open.*

eTable 7. McNemar Test Results for Pairwise Model Comparisons Using Self-Concept Narratives

Depression detection	χ^2	*p* value[a]
ZS LLM1 vs. ZS LLM2	0.000	1
ZS LLM1 vs. ZS LLM3	64.89	<.001
ZS LLM1 vs. FS LLM1	23.78	<.001
ZS LLM1 vs. FS LLM2	0.162	.69
ZS LLM1 vs. FS LLM3	9.316	.002
ZS LLM2 vs. ZS LLM3	62.95	<.001
ZS LLM2 vs. FS LLM1	21.36	<.001
ZS LLM2 vs. FS LLM2	0.165	.69
ZS LLM2 vs. FS LLM3	8.752	.003
ZS LLM3 vs. FS LLM1	19.59	<.001
ZS LLM3 vs. FS LLM2	67.35	<.001
ZS LLM3 vs. FS LLM3	38.09	<.001
FS LLM1 vs. FS LLM2	24.34	<.001
FS LLM1 vs. FS LLM3	4.808	.028
FS LLM2 vs. FS LLM3	8.007	.005
**Suicide risk detection**	χ^2	*p* value[a]
ZS LLM1 vs. ZS LLM2	1.520	.22
ZS LLM1 vs. ZS LLM3	20.35	<.001
ZS LLM1 vs. FS LLM1	0.643	.42
ZS LLM1 vs. FS LLM2	1.700	.19
ZS LLM1 vs. FS LLM3	4.190	.041
ZS LLM2 vs. ZS LLM3	8.461	.004
ZS LLM2 vs. FS LLM1	0.179	.67
ZS LLM2 vs. FS LLM2	0.011	.92
ZS LLM2 vs. FS LLM3	0.720	.40
ZS LLM3 vs. FS LLM1	11.56	.001
ZS LLM3 vs. FS LLM2	6.812	.009
ZS LLM3 vs. FS LLM3	5.047	.025
FS LLM1 vs. FS LLM2	0.350	.55
FS LLM1 vs. FS LLM3	1.901	.17
FS LLM2 vs. FS LLM3	0.519	.47

Abbreviations: AUROC, area under the receiver operating curve; CI, confidence interval; FS, few-shot; LLM1, GPT-4o (May 13, 2024, version; OpenAI); LLM2, gemini-1.0-pro (February 2024 version; Google); LLM3, GPT-3.5-turbo-16k (January 25, 2024, version; OpenAI); ZS, zero-shot.

[a]Statistically significant at $p < 0.0033$ after Bonferroni correction for multiple comparisons ($\alpha = 0.05/15$).

© 2025 Lho SK et al. *JAMA Network Open.*

eTable 8. Statistically Significant Mean AUROC Differences and 95% Confidence Intervals Between Model Pairs for Embedding-Based ML Models Using Self-Concept Narratives

Depression detection[a]	Mean AUROC difference	95% CI lower	95% CI upper
Text-embedding 1 XGB vs Text-embedding 2 SVM	0.215	0.064	0.365
Text-embedding 1 XGB vs Text-embedding 2 LR	0.199	0.050	0.349
Text-embedding 1 XGB vs Text-embedding 2 NN	0.142	0.004	0.281
Text-embedding 1 XGB vs Text-embedding 3 SVM	0.200	0.055	0.346
Text-embedding 1 XGB vs Text-embedding 3 LR	0.184	0.038	0.332

Abbreviations: AUROC, area under the receiver operating curve; CI confidence interval; LR, logistic regression; SVM, Support vector machine; text-embedding 1, text-embedding-3-large (OpenAI); text-embedding 2, text-embedding-3-small (OpenAI); text-embedding 3, text-embedding-002-ada (OpenAI); XGB, Extreme gradient boosting.

[a]Only comparisons among the 66 x 2 pairs (for depression and suicide) with statistically significant AUC differences (where the 95% CI does not include zero) are presented.

© 2025 Lho SK et al. *JAMA Network Open.*

eMethods. Procedures for Qualitative Analysis

We selected three cases including four narratives to illustrate the factors that may have influenced the model performance. One patient with clinically significant depression was identified as depressed by most large language models (LLMs) based on self-concept narratives but not as depressed based on the gender perception narratives. Two patients who had clinically significant depression based on self-concept narratives, however, were not detected as depressed by most LLMs.

We modified the prompt in Table 1 to reveal the reasoning process of GPT-4o. We passed each narrative along with the prompt to GPT-4o, obtaining both the reasoning and prediction of depression. We used Python for the analysis. The prompt we used is as follows: *"You are an experienced psychiatrist specializing in diagnosing and treating depression. I will provide you with narratives from a participant's sentence completion test (SCT). Your task is to decide whether the participant is at high risk of depression (Label 1) or lower risk of depression (Label 0). Please provide reasoning."*

After we obtained the reasoning from GPT-4o, we examined thematic content in both the original narratives and reasoning output, and overall psychological characteristics to identify factors that had influenced model performance. Two researchers independently reviewed the narratives and then reached consensus on key patterns.

© 2025 Lho SK et al. *JAMA Network Open.*

eTable 9. Narratives and GPT-4o Reasoning in Qualitative Analysis

Case 1 (self-concept narratives), BDI 50: clinically significant depression, GPT-4o prediction: High risk of depression	
Concatenated narratives[a]	I am enduring every single day. My good points are that I am quick to notice things. My biggest problem is that I am too sensitive. My abilities are learning and understanding things quickly. Someday I will leave without anyone knowing.<br>To be truly happy, I need to figure out what makes me happy. What I want most is something I am unsure of. When I was young, people said I used to laugh a lot.<br>If I could be young again, I would have learned a skill. When I get old, I am afraid I will become lonely. When I get irritated, I can't sleep. What I want to forget at all costs is the memory of my past lover, who led to my hospitalization. What bothers me is that I might be the one causing my own suffering. I feel guilty about meeting people without much thought. When things don't go as planned, I just let it be.<br>When something strange happens to me, I take a tranquilizer.
GPT-4o reasoning[b]	Expressions of endurance and uncertainty: The participant mentions "I am enduring every day," which suggests a sense of struggle and possibly a lack of enjoyment or fulfillment in daily life. Additionally, the statement "I don't know how to be truly happy" indicates uncertainty about achieving happiness, which is a common feature in depression.<br>Negative self-perception and sensitivity: The participant identifies their sensitivity as a problem ("My biggest problem is that I am too sensitive"), which may contribute to emotional distress and vulnerability to depressive symptoms.<br>Thoughts of disappearing: The statement "Someday I will leave without anyone knowing" could be interpreted as a passive expression of suicidal ideation or a desire to escape, which is a significant risk factor for depression.<br>Fear of loneliness: The fear of becoming lonely in old age ("When I get old, I am afraid I will become lonely") suggests a preoccupation with isolation, which can be both a symptom and a risk factor for depression.<br>Sleep disturbance: The participant mentions difficulty sleeping when irritable ("When I get irritated, I can't sleep"), which is a common symptom of depression.<br>Past trauma and regret: The desire to forget memories of a past relationship that led to hospitalization ("What I want to forget at all costs is the memory of my past lover, who led to my hospitalization") indicates unresolved trauma and emotional pain, which are associated with depression.<br>Self-criticism and guilt: The participant expresses guilt over indiscriminate social interactions ("I feel guilty about meeting people without much thought"), which suggests self-criticism and potential low self-esteem.<br>Reliance on medication: The mention of seeking tranquilizers when something strange happens ("When something strange happens to me, I take a tranquilizer") indicates reliance on medication to cope with stress or anxiety, which can be indicative of underlying depression.
Case 1 (gender perception narratives), BDI 50: clinically significant depression, GPT-4o prediction: Lower risk of depression	
Concatenated narratives[a]	When people talk about sex, it's interesting. The kind of woman I desire is kind and intelligent. When I see a pregnant woman, I want to help her. My wife (or husband) should be kind. The ideal man is responsible and healthy. Women are lucky because I don't know. I think men are hard to understand. Men are lucky because they are born with healthy bodies. For a woman to have a career is admirable. About married life, I feel like I want to get married soon. What I dislike most about men is nothing. I think women are someone who should be protected. The biggest disadvantage of being a woman is having a weak body. When I'm with friends of the opposite sex, I feel nervous. Men think about women as difficult to understand.
GPT-4o reasoning[b]	Based on the provided sentence completion test (SCT) responses, the participant seems to hold traditional views on gender roles, expressing a desire for partners to be kind and intelligent, and perceiving women as needing protection. The participant also expresses a desire to help pregnant women and views women having careers positively. There is no explicit mention of depressive symptoms

© 2025 Lho SK et al. *JAMA Network Open.*

	such as hopelessness, worthlessness, or persistent sadness. The participant's responses do not indicate significant negative self-perception or pervasive negative thoughts, which are often associated with high risk of depression.
Case 2 (self-concept narratives), BDI 44: clinically significant depression, GPT-4o prediction: Lower risk of depression	
Concatenated narratives[a]	I am a person who is considerate of others, works hard, and sees things through to the end once I become deeply involved. My good points are that I take care of small details and naturally show kindness and respect. My biggest problem is that I have a sense of inferiority that I don't outwardly express, which makes things difficult for me. My abilities are that I can always manage to be at least average wherever I go. Someday I will have a stable family and a steady income, and I want to live a life of helping and serving others. To be truly happy, I need to take actions that build my confidence, and I believe that financial stability and a happy family would bring me happiness. What I want most is to become someone my parents don't have to worry about. When I was young, I grew up without much hardship and enjoyed playing computer games more than studying. If I could be young again, I would read a lot of books. When I get old, I want to read a lot of books. When I get irritated, I hold it in and try to leave the situation. What I want to forget at all costs is nothing, because I believe my past has made me who I am today. What bothers me is that I have disappointed people who trusted me.<br>I feel guilty about passing my responsibilities onto others. When things don't go as planned, I feel disappointed and reflect on why things didn't work out. When something strange happens to me, I seek advice from the people around me.
GPT-4o reasoning[b]	Positive self-perception: The participant describes themselves as considerate, diligent, and action-oriented. They recognize their strengths, such as being attentive to details and having a sense of etiquette and consideration for others.<br>Future aspirations and goals: The participant expresses clear and positive future aspirations, such as having a stable family and income, and living a life of service to others. This indicates a forward-looking perspective and a sense of purpose.<br>Coping mechanisms: When faced with anger, the participant mentions trying to control it by leaving the situation, which suggests a level of emotional regulation.<br>Reflection and learning: The participant reflects on past experiences and expresses a desire to learn and grow, such as wanting to read more books if they were younger. This indicates a growth mindset.<br>Social support and problem-solving: The participant seeks advice from others when encountering unusual situations, suggesting they have a support network and are open to external input.<br>Acknowledgment of weaknesses: The participant is aware of their feelings of inadequacy and the tendency to hide them, but this self-awareness can be a protective factor against depression as it allows for personal growth and improvement.<br>Absence of hopelessness or helplessness: There is no indication of hopelessness, helplessness, or pervasive negative thoughts, which are often associated with higher risk of depression.<br>Overall, the participant demonstrates self-awareness, a positive outlook on the future, and effective coping strategies, which are protective factors against depression.
Case 3 (self-concept narratives), SDS 65: clinically significant depression, GPT-4o prediction: Lower risk of depression	
Concatenated narratives[a]	I am living my life in my own way. My good point is that I am calm. My biggest problem is that I have to take the college entrance exam next year. My abilities are that I eat well and sleep well. Someday I will do well. To be truly happy, I'm not sure what I need. What I want most is to get accepted into college. When I was young, I should have played more. If I could be young again, I would meet up with my close friends more often. When I get old, I think I will feel sad. When I get irritated, I swear. What I want to forget at all costs are embarrassing memories. What bothers me is the college entrance exam. I feel guilty about wasting a day without meaning. When things don't go as planned, I remind myself that it's nothing new and it happens. When something strange happens to me, I

© 2025 Lho SK et al. *JAMA Network Open.*

	sometimes think that maybe I am a special person.
GPT-4o reasoning[b]	Positive self-perception: The participant describes themselves as "living in their own way" and highlights a positive trait of being "calm." This suggests a level of self-acceptance and self-awareness, which are generally protective factors against depression.<br>Future orientation and optimism: The participant expresses a belief that "someday I will be fine" and has a clear goal of "getting into college." This indicates a sense of hope and future orientation, which are typically associated with lower depression risk.<br>Acknowledgment of stressors: The participant identifies the college entrance exam as a significant stressor, which is a common concern for students in South Korea. However, they do not express overwhelming despair or hopelessness about it, suggesting they are managing this stressor.<br>Regret and reflection: The participant reflects on their past with some regret, saying "I should have played more when I was young," and expresses a desire to reconnect with friends if they were younger. While these reflections indicate some dissatisfaction, they are not unusual and do not necessarily indicate depression.<br>Coping mechanisms: The participant mentions using profanity when irritated and wanting to forget "embarrassing memories," which are common coping mechanisms. They also express a sense of resilience by acknowledging that not everything goes as planned but accepting it as a part of life.<br>Lack of severe symptoms: There is no mention of severe symptoms such as persistent sadness, lack of interest in activities, or feelings of worthlessness, which are more indicative of high-risk depression.<br>Overall, while the participant experiences stress and some regret, their responses suggest they have a generally positive outlook, future goals, and coping strategies, indicating a lower risk of depression.

[a]These are the translated versions of the Korean narratives. The original Korean narratives were provided to the model during the analysis.
[b]The GPT-4o reasoning output was partly in English (the model's reasoning) and partly in Korean (quotation from the narratives). We translated the quoted narratives into English.

© 2025 Lho SK et al. *JAMA Network Open.*

eResults. Case Summaries of Qualitative Analysis

Case 1, who had a BDI score of 50, showed a clear contrast between different narrative types. The self-concept contained negative self-image and pessimistic thoughts about the future, while the gender perception narratives showed no signs of distorted beliefs about gender roles. This suggests that narrative types, particularly those reflecting an individuals' perception of self, future, and the world, might be more suitable for detecting depression.

Case 2 had a BDI score of 44, indicating clinically significant depression. However, the patient's responses were generally positive in tone. We examined the detailed psychological profile and understood that the patient had a tendency toward defensiveness and difficulty with self-disclosure. The patient's defensive response style, attempting to show a positive image of oneself, may have masked the true emotional state in the SCT narratives.

Case 3 had an SDS score of 65, indicating clinically significant depression. However, the responses were superficial. Although the patient had high average intelligence, suggesting no cognitive impairment, the psychological profile indicated a lack of depth in thinking patterns. This limited self-reflection likely resulted in consistently superficial responses in the SCT, leading to failed detection of depression based on narratives alone.

© 2025 Lho SK et al. *JAMA Network Open.*
