# Supplementary material for: Large Language Models and Text Embeddings for Detecting Depression and Suicide in Patient Narratives
Source: JAMA Netw Open. 2025 May 23;8(5):e2511922. doi: 10.1001/jamanetworkopen.2025.11922 (PMC12102709; doi:10.1001/jamanetworkopen.2025.11922)
Supplement: Supplement 2. — Data Sharing Statement [file jamanetwopen-e2511922-s002.pdf]

Data Sharing Statement

Lho. Large Language Models and Text Embeddings for Detecting Depression and Suicide in Patient Narratives. *JAMA Netw Open*. Published May 22, 2025.
doi:10.1001/jamanetworkopen.2025.11922

Data
Data available: No

Additional Information
Explanation for why data not available: The data are not publicly available due to institutional regulations. Data may be available upon reasonable request to the corresponding author, but only after approval from the institution. The code used for the analysis is available upon reasonable request to the corresponding author.
